# Effectiveness of guideline-based care by occupational physicians on the return-to-work of workers with common mental disorders: design of a cluster-randomised controlled trial

**DOI:** 10.1186/1471-2458-13-193

**Published:** 2013-03-06

**Authors:** Karlijn M van Beurden, Evelien P M Brouwers, Margot C W Joosen, Berend Terluin, Jac J L van der Klink, Jaap van Weeghel

**Affiliations:** 1 Department of Social and Behavioral Sciences, Tranzo Scientific Center for Care and Welfare, Tilburg University, PO Box 90153, Tilburg, 5000 LE, The Netherlands; 2 EMGO Institute for Health and Care Research, Department General Practice, VU University Medical Center Amsterdam, PO Box 7057, Amsterdam, 1007 MB, The Netherlands; 3 Department of Health Sciences, Work & Health, University Medical Center Groningen, University of Groningen, A. Dreusinglaan 1, Groningen, 9713 AV, The Netherlands

**Keywords:** Common mental disorders, Sick leave, Return-to-work, Occupational health service, Occupational physicians, Guideline adherence, Guideline-based care

## Abstract

**Background:**

Sickness absence due to common mental disorders (such as depression, anxiety disorder, adjustment disorder) is a problem in many Western countries. Long-term sickness absence leads to substantial societal and financial costs. In workers with common mental disorders, sickness absence costs are much higher than medical costs. In the Netherlands, a practice guideline was developed that promotes an activating approach of the occupational physician to establish faster return-to-work by enhancing the problem-solving capacity of workers, especially in relation to their work environment. Studies on this guideline indicate a promising association between guideline adherence and a shortened sick leave duration, but also minimal adherence to the guideline by occupational physicians. Therefore, this study evaluates the effect of guideline-based care on the full return-to-work of workers who are sick listed due to common mental disorders.

**Methods/design:**

This is a two-armed cluster-randomised controlled trial with randomisation at the occupational physician level. During one year, occupational physicians in the intervention group receive innovative training to improve their guideline-based care whereas occupational physicians in the control group provide care as usual. A total of 232 workers, sick listed due to common mental disorders and counselled by participating occupational physicians, will be included. Data are collected via the registration system of the occupational health service, and by questionnaires at baseline and at 3, 6 and 12 months. The primary outcome is time to full return-to-work. Secondary outcomes are partial return-to-work, total number of sick leave days, symptoms, and workability. Personal and work characteristics are the prognostic measures. Additional measures are coping, self-efficacy, remoralization, personal experiences, satisfaction with consultations with the occupational physician and with contact with the supervisor, experiences and behaviour of the supervisor, and the extent of guideline adherence.

**Discussion:**

If the results show that guideline-based care in fact leads to faster and sustainable return-to-work, this study will contribute to lowering personal, societal and financial costs.

**Trial registration:**

ISRCTN86605310

## Background

Sickness absence due to common mental disorders (CMD), such as depression, anxiety disorder and adjustment disorder, is a problem in many Western countries, including Sweden, Germany, the UK and the Netherlands [1]. Moreover, CMD have negative consequences for the worker. They affect functioning in private life and can lead to long-term absenteeism, which is associated with individual suffering, reduced probability of eventual return-to-work (RTW), a weakened financial position, social isolation, and exclusion from the labour market [2,3]. Only 50% of the workers sick listed for 6 months or more return to their work [4]. In workers with CMD, sickness absence costs are reported to be much higher than the medical costs, mainly due to the long duration of a sick leave period [5,6]. In addition, (long-term) sickness absence leads to substantial social and financial costs for society [3]. In the Netherlands, about one third of people receiving disability benefits do so because of mental health problems [7,8] of which most are CMD [7]. The annual costs of sickness absence due to CMD are estimated at 2.7–7.5 billion euros [6,9].

In 2000, the Netherlands Society of Occupational Medicine (NVAB) developed a practice guideline entitled ‘The management of mental health problems of workers by occupational physicians’ and revised it in 2007 [10,11]. This guideline, which is both practice and evidence-based, promotes an activating approach by the occupational physician (OP) aimed to establish faster RTW by enhancing the problem-solving capacity of workers, especially in relation to their work environment [7]. The guideline was disseminated among Dutch occupational health services (OHS) and OPs. In addition, educational meetings were organised (nationally and locally) for OPs to increase their knowledge on the guideline content. The OPs themselves and the OHS are expected to obtain the required skills to perform in accordance with the guideline. However, a retrospective study showed that the quality of the occupational care provided did not fully meet the requirements of the guideline, and that in workers with adjustment disorders closer adherence to this guideline was associated with a shortened sick leave duration [12]. Another Dutch study provided OPs with a three-day training in guideline use; results showed that, although their compliance was minimal, OPs had a positive attitude towards using the guideline [6,13]. Therefore, present study investigates whether guideline adherence leads to faster and sustainable RTW of workers with CMD.

### Aim of this study

To evaluate the effect of guideline-based care by OPs on the full RTW of workers sick listed due to CMD.

## Methods/design

In describing the design of this study the CONSORT 2010 statement was followed to improve the reporting quality for randomized controlled trials (RCT) [14].

### Study design

**Figure 1:**
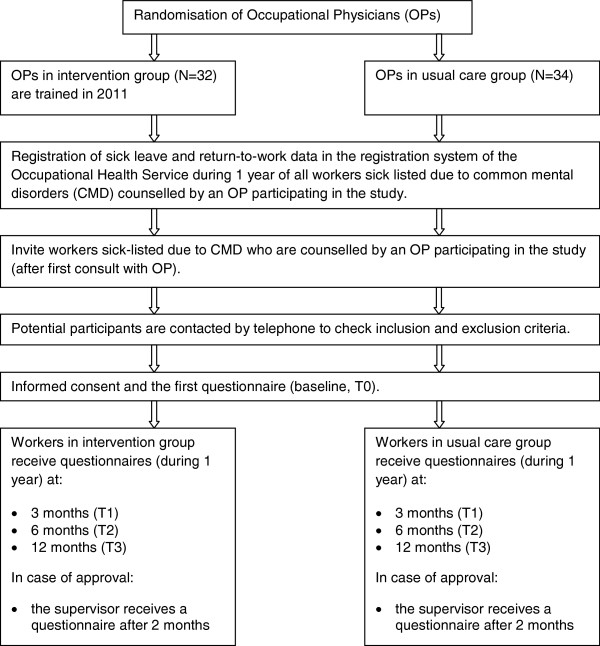
Flow diagram of the study design.

The study is designed as a two-armed cluster RCT with randomisation at the OP level (Figure 1). All participating OPs are recruited from a large collaborating OHS in the Netherlands. The OPs are randomised to an intervention group or a control group. Using an innovative training, OPs in the intervention group are trained to counsel sick-listed workers according to the Dutch national guideline ‘Management of mental health problems of workers by occupational physicians’. OPs in the control group receive no training and counsel sick-listed workers with care as usual. Workers are invited by the OHS after their first meeting with the OP. Data on sick leave and RTW of all invited workers are anonymously extracted from the registration system of the OHS during 1-year follow-up. In addition, in case of consent the worker receives a questionnaire at baseline (T0), and at 3 months (T1), 6 months (T2) and 12 months (T3) post baseline. In addition, 2 months after baseline a short questionnaire is sent to their supervisor.

The Medical Research Ethics Committee of Elisabeth Hospital in Tilburg approved the study design, protocol, information letter and brochure, questionnaires, and informed consent. Participation of workers is voluntary and all participants signed an informed consent. Each participant was informed about their right to withdraw from the study at any time.

### Intervention

#### Intervention/guideline-based care

The Dutch guideline ‘The management of mental health problems of workers by occupational physicians’ promotes an activating approach of the OP as case and care manager to enhance the problem-solving capacity of the workers to achieve RTW. The guideline is based on cognitive behavioural principles to enhance the problem-solving capacity of workers in relation to their work environment, and process-based evaluation.

The guideline consists of four consecutive steps:

1) Problem orientation and Diagnosis: an early involvement of the OP is promoted (first assessment and start of counselling about 2 weeks after the worker reported sick). A simplified classification of mental health problems is introduced in four categories: i) Stress-related complaints, ii) depression, iii) anxiety disorder, and iv) other psychiatric disorders. Furthermore, problem inventory should focus on factors related to the worker and their work environment and the interaction between these two.

2) Intervention/Treatment: the OP acts as case manager by monitoring and evaluating the process of recovery (process-based evaluation). When recovery stagnates OPs should intervene by acting as care manager by using cognitive behavioural techniques to enhance the problem-solving capacity of the worker, providing the worker and work environment with information/advice on the recovery and the RTW process, contact the general practitioner when problems remain the same or increase, and refer the worker to a specialised intervention when necessary. In addition, the OP should advise the work environment (e.g. supervisors, managers, human resource managers) how to support the worker and enhance the recovery and RTW process.

3) Relapse prevention: Integration of relapse prevention from the first contact with the worker by enhancing the problem-solving capacity of the worker.

4) Evaluation: During follow-up meetings evaluation of the recovery process includes the perspectives of the worker, supervisor, and other involved professionals. Follow-up meetings with the worker should take place every 3 weeks during the first 3 months, and then every 6 weeks thereafter. The supervisor or work environment should be contacted once a month. Follow-up contacts with the general practitioner or other professionals should take place when the recovery process stagnates or when there is doubt about the diagnosis or treatment.

#### Content of the training

OPs participating in the study and allocated to the intervention group received training in the guideline before the start of the study. This training was specifically designed for the purpose of this study and consisted of 8 meetings within 12 months. Each meeting took 2 h and was provided in groups of 4-6 OPs under the guidance of a trainer. The aim of the training was to enhance guideline adherence of the participating OPs by focussing on barriers that prevent OPs from using the guideline and finding solutions to overcome these barriers. This is considered to be a successful strategy for the implementation of guidelines [15,16].

During the 8 meetings the (key) recommendations within each consecutive step of the guideline were discussed. These discussions first focussed on barriers that would hinder OPs from using the specific recommendation in practice. The analysis of barriers was structured and based on an existing framework of barriers [17]. According to this framework guideline adherence can be affected by three main categories: 1) knowledge-related barriers (lack of awareness and lack of familiarity), 2) attitude-related barriers (lack of agreement, lack of self-efficacy, lack of outcome expectancy and lack of motivation/inertia of previous practice) and 3) external barriers that hinder physicians to apply the guideline in practice (guideline factors, environmental factors and patient factors). Second, the OPs in the group were invited to suggest solutions to address the perceived barriers taking into account the context of their daily practice. Third, the OPs drew up an action plan of how to implement these solutions in their daily practice, and agreed on learning objectives and ‘homework’ assignments. Between the meetings (a period of about 6 weeks) the OPs practiced the suggested solutions to experience if and how these would help to apply the guideline recommendations. During the next meeting the experiences of the OPs were evaluated and, when necessary, the solutions were adjusted to what the OPs had experienced in practice. This cycle of plan-do-check-act was repeated in each meeting for all the recommendations stated in the guideline.

#### Care as usual

The OPs in the control group do not receive additional training. They provide care as usual to workers on sick leave. In the Netherlands this means that the OP guides the sick-listed worker during sickness absence, recovery, and RTW. In this process the OP makes a diagnosis, assesses the ability to work, gives advice on work adaptations to the worker and the work environment, and provides relapse prevention. Although OPs are expected to work in accordance with the Dutch guideline, their actual adherence is low [12,13].

The extent of guideline adherence of the participating OPs will be measured by auditing the medical records of workers.

### Recruitment of OPs

All 66 participating OPs were recruited between October 2010 and January 2011 from the collaborative OHS. A researcher presented the study during OP meetings at several agencies of the OHS, provided written information about the study, and provided a registration form and informed consent. OPs participated on a voluntary basis; after completing the training the OPs in the intervention group received educational credits.

### Recruitment of participants

The study is conducted in the southern part of the Netherlands. The workers eligible for this study are on sick leave due to CMD diagnosed by the OP, counselled by an OP participating in the study, have had a first meeting with the OP, and are aged 18–65 years. From all eligible workers data on sick leave and RTW will be extracted from the registration system of the OHS anonymously.

Workers eligible for the data collection by means of the questionnaires work at one of the ± 320 companies served by this collaborating OHS that gave permission to invite their workers. The companies vary in size and serve different sectors. These workers are selected from the registration system of the OHS after their first consultation with the OP. They are sent an invitation letter from the OHS, as well as written information about the study. Workers who do not want to be contacted further can indicate this on a reply card. All eligible participants are contacted for additional information, and to check the inclusion and exclusion criteria. If the worker is willing to participate in the study and meets all the selection criteria, an informed consent and the baseline questionnaire will be sent to the worker.

Inclusion criteria for this study are: 1) CMD is the primary reason for sick leave diagnosed by an OP according to the Dutch Classification of Diseases (CAS) which is based on the ICD-10, 2) on current sick leave when selected from the registration system of the OHS after the first meeting with the OP, and 3) adequate command of the Dutch language. Exclusion criteria are: being suicidal, and a physical problem being the primary reason for sick leave at the time of study inclusion.

### Outcomes

Table 1 presents an overview of the collected data and the study time path.

**Table 1:** Collection of data and time path

**Topic**	**Instrument**	**Baseline**	**Follow-up**		
		**T0**	**T1**	**T2**	**T3**
			**3 months**	**6 months**	**12 months**
**Primary outcome**					
Full RTW	Registration system of the occupational health care service	X	X	X	X
**Personal characteristics**					
Gender, age, level of education, diagnosis by OP, sick leave in the previous year, history of mental disorders, expectations about full RTW.		X			
**Work characteristics**					
Type of function, number of working hours, contract type.		X			
Job content	JCQ	X	X	X	X
**Secondary outcomes**					
Partial RTW	Registration system of the occupational health care service	X	X	X	X
Total numbers of sick leave days	Registration system of the occupational health care service	X	X	X	X
CMD symptoms	4DSQ	X	X	X	X
Burnout symptoms	UBOS	X	X	X	X
Workability	3 questions of WAI	X	X	X	X
**Additional outcomes**					
Coping	Shortened 19-item version UCL	X	X	X	X
Self-efficacy	RTW-SE	X	X	X	X
Remoralization	RS-12	X	X	X	X
Experienced barriers, facilitators and social support for RTW		X	X	X	X
Experience and satisfaction with OP	Adapted version PSOHPQ	X	X	X	X
Experience and satisfaction with supervisor		X	X	X	X
**2 months after inclusion**					
Experiences supervisor					
Contact with worker, sick leave worker, work adaptations, contact with OP, CMD and sick leave, policy on sick leave and RTW			X		
Personal characteristics supervisor			X		

#### Primary outcome

The primary outcome is the time to full RTW. For this purpose the number of calendar days between the first day of sickness absence due to CMD and the first day of full RTW is calculated. Working the same hours as prior to the sickness absence in own or equivalent work for at least 4 weeks is considered full RTW. This means that reporting sick within 4 weeks of full RTW is not considered as a new period of sickness absence.

#### Secondary outcomes

•Partial RTW is defined as the number of calendar days between the first day of sickness absence due to CMD and the first day of RTW, irrespective of the number of working hours per week.

•Total number of calendar days of sick leave is calculated for the 1-year follow-up period.

•CMD symptoms are measured by the Four Dimensional Symptoms Questionnaire (4DSQ), a self-report questionnaire measuring the four dimensions of common psychopathology: distress, depression, anxiety and somatisation. The 4DSQ consists of 50 items (each scored on a 5-point scale) and refers to symptoms during the past week. The 4DSQ has good psychometric properties [18].

•Burnout symptoms are measured by the Utrechtse Burnout Scale-General Survey (UBOS) [19], which is the Dutch version of the Maslach Burnout Inventory (MBI). The UBOS is a self-report questionnaire which measures three subscales: emotional exhaustion, mental distance, and competence. Higher scores on exhaustion and distance and lower scores on competence indicate burnout. The UBOS is a reliable and valid instrument [20].

•Workability is measured by three questions (items 1, 2, 3) of the shortened version of the Work Ability Index (WAI) [21,22]. The WAI is a self-report questionnaire and is a reliable and valid instrument [23,24].

#### Prognostic measures

•Personal characteristics such as age, gender, level of education, sick leave in the previous year, history of mental disorders, and expectations about full RTW are measured at baseline.

•Work characteristics such as number of working hours, contract type, type of work, profession and job content are measured at baseline. Job content is measured with the Dutch version of the Job Content Questionnaire (JCQ) [25], a self-report questionnaire which measures the social and psychological characteristics of jobs. The JCQ assesses the following scales: psychological job demands, decision latitude, social support, physical demands and job insecurity.

#### Additional measures

Factors which can be influenced by the intervention and thereby can influence RTW are also measured. The results of these additional measures will be reported separately from the results of this RCT.

•Coping style is measured with the shortened 19-item version of the Utrecht Coping List (UCL) [26], a self-report questionnaire which measures coping behaviour. The 19-item version assesses the following scales: 1) active problem solving, 2) seeking social support, 3) palliative reaction pattern, 4) avoidance behaviour, and 5) expression of emotions.

•Self-efficacy with regard to RTW is measured by the RTW-SE for workers with mental problems. The RTW-SE is a self-report questionnaire which assesses the self-efficacy in the RTW process. The RTW-SE shows promising reliability, validity and prediction of actual RTW within 3 months [27].

•Remoralization (perception of recovery) is measured with the 12-item Remoralization Scale (RS-12). The RS-12 is a self-report questionnaire which indicates the level of morale in mental health care and has shown promising reliability and validity [28].

•Workers’ experiences with the consultations with their OP, and the contact with their supervisor, are measured. For example, the number and content of the consultations, and the topics of the conversations. Satisfaction with the counselling by the OP is measured with an adapted version of the Patient Satisfaction with Occupational Health Professionals Questionnaire (PSOHPQ) [29].

•Experiences of the supervisor are measured using self-formulated questions. This questionnaire includes topics such as the contact with worker and the OP, previous experience with CMD and sick leave in general, and policy on sick leave and RTW [see Additional file 1].

### Sample size

A power analysis was performed to determine the sample size needed to detect a difference between the control and intervention group with respect to the time workers fully return to their work (primary outcome measure). Proportions of full RTW were adopted from previous studies [7,30]. It was assumed that in the usual care (control) group 55% of the workers would have returned to work after 3 months and 75% after 6 months, whereas in the intervention group these figures would be 75% and 90%, respectively. With a power of 80% at a 0.05 alpha level, assuming an ICC of 0.025 and taking into account a correction factor for the clustered design, it was calculated that 2 × 97 workers would be needed to detect the difference after 3 months and 2 × 110 workers for the difference after 6 months. Allowing for 5% attrition on the sick leave data, a total of 232 workers need to be included.

### Randomisation

Randomisation takes place at the OP level, because workers cannot be randomly allocated to an OP in the intervention group or an OP in the control group since every OP is allied to a specific company. All participating OPs are randomised by computerised allocation to the intervention group or control group at OHS agency level.

### Blinding

Workers and companies are blinded for randomisation since they are not aware of the allocation of their OP. The researcher who performs the analyses (KvB) is blinded for allocation to intervention or care as usual.

### Statistical analysis

Survival analyses will be used to analyse the primary outcome (time to full RTW) and the time to partial RTW comparing the intervention and the control group, while taking into account the effect of clustering of workers within OPs. Longitudinal multilevel analysis will be used to analyse the secondary outcomes.

To detect significant differences in the baseline characteristics between the intervention group and control group descriptive analyses will be used. If necessary these differences will be taken into account in the effect evaluation.

## Discussion

The societal relevance of this study consists of substantial personal, social and financial savings if guideline-based care leads to faster and sustainable RTW of workers with CMD. Since previous studies indicate that guideline adherence of OPs can lead to a shortened sick leave duration [6,12,13], the training described in this study aims to improve the OPs’ guideline adherence. Therefore, the present study evaluates the effect of guideline-based care on full RTW of workers with CMD.

### Strengths and limitations

A strength of this study is the collaboration with one of the largest OHS in the Netherlands; this provides a diversity of companies covering many sectors, yielding a heterogeneous population which allows to generalise the results to a larger working population. Furthermore, because the innovative training is spread over one year, OPs can explore the barriers, apply solutions, evaluate their experiences, and adapt the solutions until they are useful in daily practice. This is in contrast to earlier studies which evaluated short term training only. Another strength is that the participating workers are selected by the registration system of the OHS and not by OPs; this may prevent selection bias from the individual OPs. Finally, the workers are blinded for randomisation to the intervention or control group to prevent performance bias.

Limitations: although the extent of guideline adherence by OPs will be measured by auditing the medical records of workers, there is a risk that this will not provide accurate information on guideline adherence: e.g. OPs might not document everything that occurred during the counselling. Another limitation might be that 232 workers are needed and followed during one year, whereas earlier studies had a problem recruiting sufficient workers. However, collaborating with one of the largest OHS in the Netherlands should ensure sufficient sick-listed workers, i.e. 60 OPs need to counsel 3-4 workers each to reach the total of 232 workers.

### Impact of study results

This study will show whether guideline-based care in fact leads to faster and sustainable RTW. If the results are promising, this study will contribute to lower societal and financial costs and less negative consequences for workers with CMD. The training may also give OPs the tools to better handle the guideline in daily practice. Moreover, the approach applied in this study may be relevant for the implementation of this and other (occupational) guidelines in daily practice. Results of this study will become available in 2015.

## Abbreviations

CMD: Common mental disorders; RTW: Return-to-work; OP: Occupational physician; OHS: Occupational health service; NVAB: The Netherlands society of occupational medicine; CAS: Dutch classification of diseases; 4DSQ: Four dimensional symptoms questionnaire; UBOS: Utrechtse burnout scale; WAI: Work ability index; JCQ: Job content questionnaire; UCL: Utrecht coping list; RTW-SE: Return-to-work self-efficacy scale; RS-12: 12-Item remoralization scale; PSOHPQ: Patient satisfaction with occupational health professionals questionnaire

## Competing interests

BT is the copyright owner of the 4DSQ and receives copyright fees from companies that use the 4DSQ on a commercial basis (the 4DSQ is freely available for noncommercial use in health care and research). BT received fees from various institutions for workshops on the application of the 4DSQ in primary care settings.

JvdK was manager and main author in the development of the NVAB guideline. JvdK does not receive fees for the use of the guideline.

KvB, EB, MJ declare that they have no competing interests.

## Authors’ contributions

EB, BT, JvdK, JvW, KvB, MJ designed the study and developed the intervention. KvB drafted the manuscript and is responsible for the data collection of the effect study. All authors provided comments on the drafts of the manuscript and approved the final version of the manuscript.

## Pre-publication history

The pre-publication history for this paper can be accessed here:

http://www.biomedcentral.com/1471-2458/13/193/prepub

## Supplementary Material

Additional file 1 An additional file shows this questionnaire for supervisors. Click here for file
