# Supplementary material for: Effectiveness of guideline-based care by occupational physicians on the return-to-work of workers with common mental disorders: design of a cluster-randomised controlled trial
Source: BMC Public Health. 2013 Mar 6;13:193. doi: 10.1186/1471-2458-13-193 (PMC3599838; doi:10.1186/1471-2458-13-193)
Supplement: Additional file 1 — An additional file shows this questionnaire for supervisors. [file 1471-2458-13-193-S1.pdf]

Vragenlijst

Herstel na ziekteverzuim en werkhervatting

Leidinggevenden

Respondentnummer:		
Datum versturen:	-	- 2012
Datum ontvangen:	-	- 2012

TILBURG UNIVERSITY

Tranzo Wetenschappelijk centrum voor zorg en welzijn

Arbo Unie omdat mensen de motor zijn

umcg

EMGO+ Institute for Health and Care Research

Introductie

Dit is de vragenlijst van het onderzoek 'Herstel na ziekteverzuim en werkhervatting'.

In deze vragenlijst staan vragen over uw contact met uw werknemer die deelneemt aan het onderzoek, zijn/haar ziekteverzuim en zijn/haar werkhervatting. In deze vragenlijst verwijzen we naar deze persoon als 'uw werknemer'.

Bij de vragen kunt uw antwoorden invullen, aankruisen of omcirkelen. Vult u steeds het antwoord in dat het meest overeenkomt met uw situatie of dat het beste bij u past. Er zijn geen goede of foute antwoorden. Het gaat om uw mening. Vul per vraag slechts één antwoord in.

Uw antwoorden worden strikt vertrouwelijk behandeld. Uw antwoorden worden uitsluitend gebruikt voor het onderzoek en worden niet verstrekt aan uw werknemer of aan uw bedrijfsarts.

Het is belangrijk dat u geen vragen overslaat.

Alvast hartelijk bedankt voor het invullen van de vragenlijst!

Datum invullen van vragenlijst (dd-mm-jjjj): …………. - ……………. - 2012

Contact met uw werknemer

De volgende vragen gaan over het contact dat u sinds de ziekmelding met uw werknemer hebt gehad.

1. Heeft u sinds de ziekmelding contact (per telefoon, per e-mail, een afspraak) gehad met uw werknemer?
   - ☐ Ja
   - ☐ Nee

2. Hoeveel contacten heeft u sinds de ziekmelding met uw werknemer gehad?
   …………... aantal

3. Hoe lang geleden was het laatste contact met uw werknemer?
   - ☐ 1 – 7 dagen geleden
   - ☐ 8 – 14 dagen geleden
   - ☐ 15 – 21 dagen geleden
   - ☐ 22 – 28 dagen geleden
   - ☐ Langer dan 4 weken geleden

4. Hoe was uw (werk)relatie met uw werknemer vóór het ziekteverzuim?
   - ☐ Slecht (conflictueus)
   - ☐ Matig
   - ☐ Redelijk
   - ☐ Goed (harmonieus)

5. Hoe is uw (werk)relatie met uw werknemer nu?
   - ☐ Slecht (conflictueus)
   - ☐ Matig
   - ☐ Redelijk
   - ☐ Goed (harmonieus)

6.	Heeft u sinds de ziekmelding met uw werknemer gesproken over de volgende onderwerpen?	Nee	Ja
A.	Zijn/haar klachten	☐	☐
B.	De afdeling waar hij/zij werkt	☐	☐
C.	De contacten op zijn/haar werk	☐	☐
D.	De beperkingen als gevolg van zijn/haar klachten	☐	☐
E.	De mogelijkheden voor werkaanpassingen	☐	☐
F.	De mogelijkheden voor werkhervatting	☐	☐
G.	Problemen ten aanzien van werkhervatting	☐	☐
H.	Oplossingen voor de problemen ten aanzien van werkhervatting	☐	☐
I.	Het realiseren van oplossingen voor de problemen ten aanzien van werkhervatting	☐	☐
J.	Een ander onderwerp, namelijk: …………………………………………………………………………	X	

7A. Heeft u uw werknemer gestimuleerd om zijn/haar werk weer (gedeeltelijk) te hervatten?

☐ Ja, ga verder met vraag 7B.
☐ Nee, ga verder met vraag 8.

7B. Hoe heeft u uw werknemer gestimuleerd om zijn/haar werk weer (gedeeltelijk) te hervatten?

………

………

………

………

8. Zijn er (tijdelijke) aanpassingen aan de werkzaamheden / taken gedaan voor uw werknemer?

☐ Ja
☐ Nee
☐ N.v.t.

9. Zijn er (tijdelijke) aanpassingen aan de werktijden van uw werknemer gedaan?

☐ Ja
☐ Nee
☐ N.v.t.

10.	Heeft u een plan van aanpak voor werkhervatting gemaakt met uw werknemer	☐ Ja<br>☐ Nee<br>☐ Nog niet
11.	Heeft uw werknemer op dit moment zijn/haar werk hervat?	☐ Ja, volledig<br>☐ Ja, gedeeltelijk<br>☐ Nee

Contact met de bedrijfsarts over deze werknemer

De volgende vragen gaan over het contact dat u hebt gehad met de bedrijfsarts over uw werknemer sinds de ziekmelding.

12.	Heeft u sinds de ziekmelding contact (per telefoon, per e-mail, een afspraak) gehad met de bedrijfsarts over uw werknemer?	☐ Ja<br>☐ Nee, ga verder met de vraag 22 / einde vragenlijst indien u de vragen 22 tot en met 43 al bij een eerdere werknemer hebt ingevuld
13.	Hoeveel contacten heeft u sinds de ziekmelding met de bedrijfsarts gehad?	………….. aantal
14.	Hoe is uw (werk)relatie met de bedrijfsarts aangaande uw werknemer?	☐ Slecht<br>☐ Matig<br>☐ Redelijk<br>☐ Goed

		Hele-maal oneens	Enigs-zins oneens	Eens noch oneens	Enigs-zins eens	Hele-maal eens	N.v.t.
15.	De bedrijfsarts ondersteunde mij om contact met mijn werknemer te blijven houden gedurende het verzuim en het werkhervattingproces.	☐	☐	☐	☐	☐	☐
16.	Met de informatie van de bedrijfsarts weet ik beter hoe het herstelproces van een werknemer met psychische klachten eruit kan zien.	☐	☐	☐	☐	☐	☐
17.	Met het advies van de bedrijfsarts weet ik beter hoe ik mijn werknemers kan ondersteunen bij zijn/haar herstel en werkhervatting.	☐	☐	☐	☐	☐	☐
18.	De bedrijfsarts leek deskundig.	☐	☐	☐	☐	☐	
19.	Over het algemeen, vond ik het contact met de bedrijfsarts over deze werknemer prettig.	☐	☐	☐	☐	☐	
20.	Over het algemeen, was het contact met de bedrijfsarts over deze werknemer nuttig.	☐	☐	☐	☐	☐	
21.	Hoe tevreden bent u, alles bij elkaar genomen, over het contact met de bedrijfsarts over deze werknemer? Geef een cijfer van 1 tot en met 10 (1 = erg ontevreden, 10 = erg tevreden).						………..

Uw ervaring met werknemers met psychische klachten en ziekteverzuim in het algemeen

De volgende uitspraken gaan over hoe u de begeleiding van werknemers met psychische klachten over het algemeen ervaart.

22. Het begeleiden van werknemers met psychische klachten vind ik over het algemeen

Erg moeilijk	Moeilijk	Neutraal	Makkelijk	Erg makkelijk

23. Het begeleiden van werknemers met psychische klachten vind ik over het algemeen

Zeer onplezierig	Onplezierig	Neutraal	Plezierig	Zeer plezierig

24. Over de manier waarop ik werknemers met psychische klachten begeleid ben ik over het algemeen

Zeer ontevreden	Ontevreden	Neutraal	Tevreden	Zeer tevreden

25. Heeft u eerder werknemers die verzuimen wegens psychische klachten gehad?
- ☐ Ja
- ☐ Nee

De volgende vragen gaan over werkhervatting en werkaanpassingen. Kruis per vraag 1 hokje aan dat het beste aansluit bij uw opvatting.

		Zelden of nooit	Soms	Vaak	Zeer vaak
26.	Gedeeltelijke werkhervatting (als eerste stap naar volledige werkhervatting) vind ik zinvol	☐	☐	☐	☐
27.	Gedeeltelijke werkhervatting (als eerste stap naar volledige werkhervatting) vind ik haalbaar	☐	☐	☐	☐
28.	Tijdelijke werkaanpassingen vind ik zinvol	☐	☐	☐	☐
29.	Tijdelijke werkaanpassingen vind ik haalbaar	☐	☐	☐	☐
30.	Permanente werkaanpassingen vind ik zinvol	☐	☐	☐	☐
33.	Permanente werkaanpassingen vind ik haalbaar	☐	☐	☐	☐
34.	Ik heb de mogelijkheid om werkaanpassingen te realiseren	☐	☐	☐	☐

Verzuimbeleid

De volgende vragen gaan over het verzuimbeleid van uw afdeling of organisatie.

35. Heeft uw afdeling of organisatie een verzuimbeleid?

- ☐ Ja
- ☐ Nee

36. Wie begeleidt normaal gesproken de werknemer tijdens het ziekteverzuim en het werkhervattingproces?

Meerdere antwoorden mogelijk

- ☐ Leidinggevende
- ☐ Bedrijfsarts
- ☐ Verzuimconsulent
- ☐ Personeelsfunctionaris
- ☐ Bedrijfsmaatschappelijk werk
- ☐ Niemand
- ☐ Weet ik niet
- ☐ Anders, nl:

…………………………………

…………………………………

38A Heeft u scholing gehad in het omgaan met werknemers met psychische klachten?

- ☐ Ja, ga naar vraag 38B en 38C
- ☐ Nee, ga naar vraag 39

38B Indien Ja, van wie heeft u scholing gekregen? ………………………………………………………………

38C Wat is de kern van wat u heeft geleerd in die scholing?

……

……

Algemene gegevens

De vragen in dit blok gaan over algemene zaken die met u of uw werk te maken hebben.

39. Wat is uw geslacht?

- ☐ Vrouw
- ☐ Man

40. Wat is uw geboortedatum? …………. - …………. – 19……..

41. Wat is uw nationaliteit?

- ☐ Nederlands(e)

- ☐ Anders, namelijk: …………………………………………………………………………………………………

42. Wat is de hoogste opleiding die u hebt voltooid?

- ☐ Geen opleiding gevolgd, afgemaakt
- ☐ Basisonderwijs
- ☐ VMBO, LBO, MAVO
- ☐ MBO
- ☐ HAVO, VWO
- ☐ HBO
- ☐ Universiteit
- ☐ Anders, namelijk: ……………………………………………………………………………………………………..

43. Wat is uw huidige werk, functie?

……

Ruimte voor opmerkingen

………

………

………

………

………

………

………

Dit is het einde van de vragenlijst.

Hartelijk bedankt voor het invullen van de vragenlijst!

U kunt de vragenlijst opsturen in de bijgevoegde antwoordenvelop naar:

Universiteit van Tilburg - Tranzo
Kamernummer: T529, T.a.v. drs. K. van Beurden
Antwoordnummer 412
5000 WB Tilburg
